# Molecular Epidemiology of Genital Infections in Campania Region: A Retrospective Study

**DOI:** 10.3390/diagnostics12081798

**Published:** 2022-07-25

**Authors:** Elena Scaglione, Giuseppe Mantova, Valeria Caturano, Luca Fanasca, Francesca Carraturo, Fabrizio Farina, Caterina Pagliarulo, Mariateresa Vitiello, Chiara Pagliuca, Paola Salvatore, Roberta Colicchio

**Affiliations:** 1 Department of Molecular Medicine and Medical Biotechnology, University of Napoli Federico II, Via S. Pansini 5, 80131 Naples, Italy; elena.scaglione@unina.it (E.S.); giuseppe.mantova@unina.it (G.M.); vale.caturano@gmail.com (V.C.); l.fanasca@studenti.unina.it (L.F.); fr.carraturo@studenti.unina.it (F.C.); mariateresa.vitiello2@unina.it (M.V.); chiara.pagliuca@unina.it (C.P.); 2 Department of Chemical, Materials and Production Engineering, University of Napoli Federico II, Piazzale V. Tecchio 80, 80125 Naples, Italy; 3 Department of Law, Economics, Management and Quantitative Methods, University of Sannio, Piazza Arechi II, 82100 Benevento, Italy; f.farina@mediastat.it; 4 Department of Science and Technology, University of Sannio, Via De Sanctis, 82100 Benevento, Italy; caterina.pagliarulo@unisannio.it; 5 CEINGE Biotecnologie Avanzate s.c.ar.l., Via Gaetano Salvatore 486, 80145 Naples, Italy

**Keywords:** genital infections, multiplex real-time PCR, epidemiology

## Abstract

This study provides updated information on the prevalence and co-infections caused by genital microorganisms and pathogens: *Mycoplasma genitalium*, *Mycoplasma hominis, Ureaplasma parvum*, *Ureaplasma urealyticum*, *Trichomonas vaginalis*, and *Gardnerella vaginalis*, by retrospectively analyzing a cohort of patients living in the Naples metropolitan area, Campania region, Southern Italy. To investigate the genital infections prevalence in clinical specimens (vaginal/endocervical swabs and urines) collected from infertile asymptomatic women and men from November 2018 to December 2020, we used a multiplex real-time PCR assay. Of the 717 specimens collected, 302 (42.1%) resulted positive for at least one of the targets named above. Statistically significant differences in genital prevalence of selected microorganisms were detected in both women (62.91%) and men (37.08%). *G. vaginalis* and *U. parvum* represented the most common findings with an 80.2% and 16.9% prevalence in vaginal/endocervical swabs and first-voided urines, respectively. Prevalence of multiple infections was 18.18% and 8.19% in women and men, respectively. The most frequent association detected was the co-infection of *G. vaginalis* and *U. parvum* with 60% prevalence. Our epidemiological analysis suggests different infection patterns between genders, highlighting the need to implement a preventative screening strategy of genital infections to reduce the complications on reproductive organs.

## 1. Introduction

Genital tract infections (GIs) remain one of the major global public health issues causing infertility, a condition that affects about 15% of couples of reproductive age, representing a globally underestimated problem with a significant psychological impact [1,2,3].

There are over 30 bacterial, viral, fungal, and parasitic pathogens known to contribute to infertility and among these human papillomavirus (HPV), *Chlamydia trachomatis*, *Ureaplasma* spp., *Neisseria gonorrhoeae*, *Trichomonas vaginalis*, and *Mycoplasma genitalium* are the most common causative agents [4,5,6]. In particular, *C. trachomatis*, *N. gonorrhoeae*, and *T. vaginalis* are classified as some of most common curable agents of sexually transmitted infections (STIs) [7,8]; recently, *M. genitalium* was recognized as an STI, albeit not a notifiable infection, and still few data exist on its prevalence [9].

GIs can cause a wide range of clinical presentations, both in the short and long term, such as urethritis, prostatitis, cervicitis, and vaginitis, which if unrecognized and left untreated may lead to relevant clinical complications including infertility, pelvic inflammatory disease, chronic pelvic pain, adverse pregnancy outcomes, premature delivery, neonatal death, and increased risk of contracting severe STI such as human immunodeficiency virus (HIV) infection [10,11,12,13,14,15,16].

It is well known that the genital tract harbors numerous microorganisms defined as microbiota that exist (in conjunction with their genes and products) in a regulated mutualistic relationship with the host [17,18]. Some of these microorganisms reinforce the defense against invasion and colonization by opportunistic pathogens [17]. Several microorganisms can infect the genital tract, but only a few can be considered as “established pathogens”, and it is still not completely understood how they interact with each other in the colonization and in the infectious processes. For this reason, additional epidemiological studies are needed to determine the significance, prevalence, and distribution of microorganisms other than recognized conventional genital pathogens in these syndromes. In addition, in many cases, GIs are asymptomatic and early detection would be useful to prevent several serious complications. Furthermore, the increasing number of asymptomatic cases leads to their under-diagnosis and possible spreading to other subjects. For example, *T. vaginalis* infection, one of the most prevalent non-viral STIs, is often underestimated due to the high frequency of asymptomatic patients contributing to the dissemination of this infection [19].

Several evidence show that a significant number of patients with an altered genital microbial balance that leads to GIs can result in co-infection with multiple pathogens at the same time [20,21,22]. For this reason, the development of methods to simultaneously detect several infections in simple steps could result fundamental for the diagnosis of GIs.

Prompt recognition and appropriate treatment are crucial for the control of GIs and their transmission and require sensitive, rapid, and accurate laboratory diagnostic assays. At the moment, the traditional methods for diagnosing GIs are laborious, with low sensitivity and a long turnaround time. Moreover, most of the organisms that cause GIs are difficult to detect with conventional microbiological strategies.

In the past few years, new molecular tools have been developed in order to improve their diagnosis and to identify a wide array of microorganisms in any clinical sample at once.

New real-time multiplex PCR (mRT-PCR) methods allow a rapid detection of genital pathogens from various specimens (endocervical swabs, vaginal swabs, and urine samples) and provide an additional advantage in screening since they detect multiple agents simultaneously from a single clinical sample with high sensitivity and specificity. Some of these specimens can be collected without the need of clinical examination, thus increasing their potential usefulness in screening strategies. However, some genital microorganisms are also common commensals in the urogenital tract of healthy people, but an increased bacterial load, evaluated by quantitative real-time PCR, can be considered an indicator of infection [23].

Moreover, several recent reports showed that genital infection screening tests using multiplex PCR assays provided higher performances than the conventional methods [24,25,26]. The important role of screening programs for GIs is demonstrated by the results reported in various studies that show a significant reduction of the incidence of these diseases [27,28].

The molecular screening could indeed reduce the risk of transmission to sexual partners leading to a reduction of treatment costs and a consequent decrease of antibiotic-resistant strains.

The purpose of the current study was to investigate the prevalence and association of co-infections of six common genital pathogens (*U. urealyticum*/*U. parvum*; *M. hominis*/*M. genitalium*, and *T. vaginalis*/*G. vaginalis*) simultaneously detected by mRT-PCR in different types of genital specimens in a cohort of patients undergoing fertility investigations at the University of Naples “Federico II” Hospital.

## 2. Materials and Methods

### 2.1. Study Population and Specimen Collection

Between November 2018 and December 2020, 717 clinical specimens from 717 subjects (363 women and 354 men) from the metropolitan area around Naples, Campania region, Southern Italy, were collected and processed by the Complex Operative Unit of Clinical Microbiology, University of Naples “Federico II” Hospital, Italy.

In particular, specimens included vaginal/endocervical swabs from female patients and first-voided urines from male patients. Sample information (date of sampling, ward, type of specimen, final testing results) together with the data of patients for whom molecular testing was performed (i.e., age and sex) were recorded in an anonymous database by changing sensitive data into alphanumeric codes. No clinical data associated with these specimens were available. Being a retrospective study, formal consent was not required.

### 2.2. Molecular Testing for GIs Detection

The targeted genital pathogens, with fastidious growth requirements or non-cultivable, were *U. urealyticum*/*U. parvum*; *M. hominis*/*M. genitalium*, and *T. vaginalis*/*G. vaginalis*, and were searched out both in first-voided urine and vaginal/endocervical swabs by multiplex real-time PCR. Vaginal/endocervical swab specimens were placed in 3 mL Amies Transport medium (Sigma Transwab, MVE medical wire, Corsham, Wiltshire, UK). The collection tubes and urine specimens were equilibrated at room temperature and mixed by vortexing, then 300 µL of samples were pre-treated with a tissue lysis buffer (ATL, QIAGEN GmbH, Hilden Germany). DNA was extracted from the specimens using the instrument EZ1 Advanced XL (QIAGEN GmbH), according to the manufacturer’s instructions, and stored frozen at −20 °C until testing. The detection of mycoplasmas, ureaplasmas, and *T. vaginalis*/*G. vaginalis* was performed with the RealLine STI Pathogen Kits (RealLine *Trichomonas vaginalis*/*Gardnerella vaginalis* kit, RealLine *Ureaplasma urealyticum*/*Ureaplasma parvum* kit, RealLine *Mycoplasma hominis*/*Mycoplasma genitalium*-BIORON Diagnostics GmbH, Romerberg, Germany), commercially available mRT-PCR assays for the detection of genital pathogens DNA. The whole process was monitored adding to each sample 20 µL of Internal Control (IC) (BIORON Diagnostics GmbH), provided by the manufacturer, before the DNA extraction, to confirm the nucleic acid extraction and to exclude PCR inhibition. The mRT-PCR tests were performed according to the manufacturer’s protocol. Briefly, the amplification was performed in a CFX96 Real-Time thermocycler (Bio-Rad, Hercules, CA, USA). Each PCR was performed with 50 µL of extracted DNA.

The thermal cycling conditions consisted of an initial incubation at 50 °C for 2 min, pre-denaturation at 95 °C for 2 min, followed by 50 cycles of alternating incubations: denaturation at 94 °C for 10 s, and annealing and extension at 60 °C for 40 s.

Samples were considered positive with an average Ct value of ≤40, except for *G. vaginalis* and *U. parvum* which were considered positive with a Ct value of ≤32, because these bacteria are normally part of the genital flora of healthy people and the positivity to the test should reflect their overgrowth more often associated with genital discharge and dysbiosis.

### 2.3. Statistical Analysis

A descriptive analysis was performed to assess the main characteristics of all the subjects enrolled in this study (number of subjects, proportion of women and men, mean age). Considering the characteristics of the variables and sample size, Chi-Square Test and Fisher’s exact non-parametric tests were used to test hypotheses about the relationship between two variables and to test differences between proportions for two populations. To measure an association with exposure, we computed the odds ratio by the classical approach (logistic regression). All tests performed were considered significant for a value of *p* < 0.05.

## 3. Results

### 3.1. Characteristics of Study Subjects

Between November 2018 and December 2020, a total of 717 subjects from the metropolitan area around Naples, Campania region in Southern Italy, were recruited for the study. Subjects must not have used antibiotics in the 15 days prior to the analysis and must not have had any type of sexual intercourse during the 48 h preceding the sample collection.

The general demographic characteristics of the enrolled subjects are presented in Table 1. In particular, the patients were 363 women (50.63%) and 354 men (49.37%) with a mean age of positive and negative for genital pathogens detection of 37.59 and 38.92, respectively.

The distribution between positive and negative cases was statistically significant among both women and men analyzed (Table 1). In detail, most women analyzed were positive to genital pathogens (*p*-value < 0.001), conversely most of the evaluated men were negative for the pathogens’ detection (*p*-value < 0.001). Amongst the positive genital pathogen detection tests (302), 190 women tested were positive with a percentage of 62.91% (190/302) compared to 112 men who tested positive with an incidence of 37.08% (112/302) (Table 1).

The retrospective analysis involved mainly patients aged between 30 and 39 (362 patients), followed by subjects ranging from 40 to 49 years (232), while the lowest number of patients were over 50 years old (55) (Supplementary Materials Table S1).

**Figure 1:**
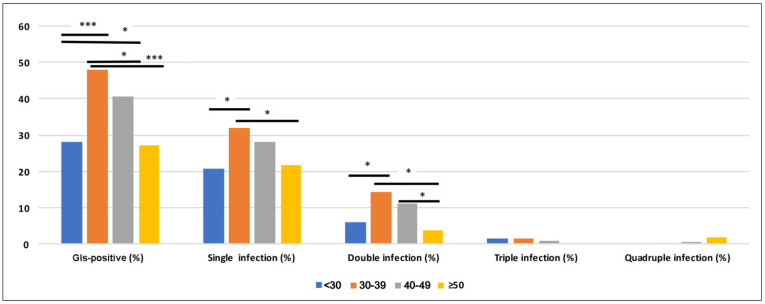
Distribution of GIs between age study groups. The statistical significance was calculated with Chi square Fisher’s exact test (*p* < 0.05, *; *p* < 0.001, ***). The risk of GIs in the age group 30–39 years was higher (Odd ratio, (O.R.): 1.66, CI: 1.23–2.24) than the other age groups analyzed. The age group 30–39 years showed a high risk for single infections (O.R. 1.98; CI: 1.45–2.71) and for double infections (O.R. 1.69; CI: 1.06–2.70) than the population analyzed.

Regarding the distribution of GIs between age study groups, patients aged 30 to 39 resulted significantly more affected compared to patients under 30 (*p*-value < 0.001) and over 50 (*p*-value < 0.001) (Figure 1). Patients of age ranging from 40 to 49 were significantly more affected compared to subjects under 30 (*p*-value < 0.05) (Figure 1). Among the six investigated microorganisms, subjects aged 30 to 39 years were the most affected group for single and double infections (*p*-value <0.05) than subjects under 30 and over 50 (Figure 1). Moreover, patients aged 40 to 49 were significantly more affected by double infections compared to subjects over 50 years old (*p*-value < 0.05) (Figure 1).

Indeed, the risk of GIs in the age group 30–39 years was higher (Odd ratio, (O.R.): 1.66, CI: 1.23–2.24) than the other age groups considered in our study. In detail, almost 50% of detected pathogens affected this age group (Supplementary Materials Table S1), with a higher risk of single infections (O.R. 1.98; CI: 1.45–2.71) and double infections (O.R. 1.69; CI: 1.06–2.70) than the population analyzed.

### 3.2. Prevalence of Single and Multiple Infections

**Figure 2:**
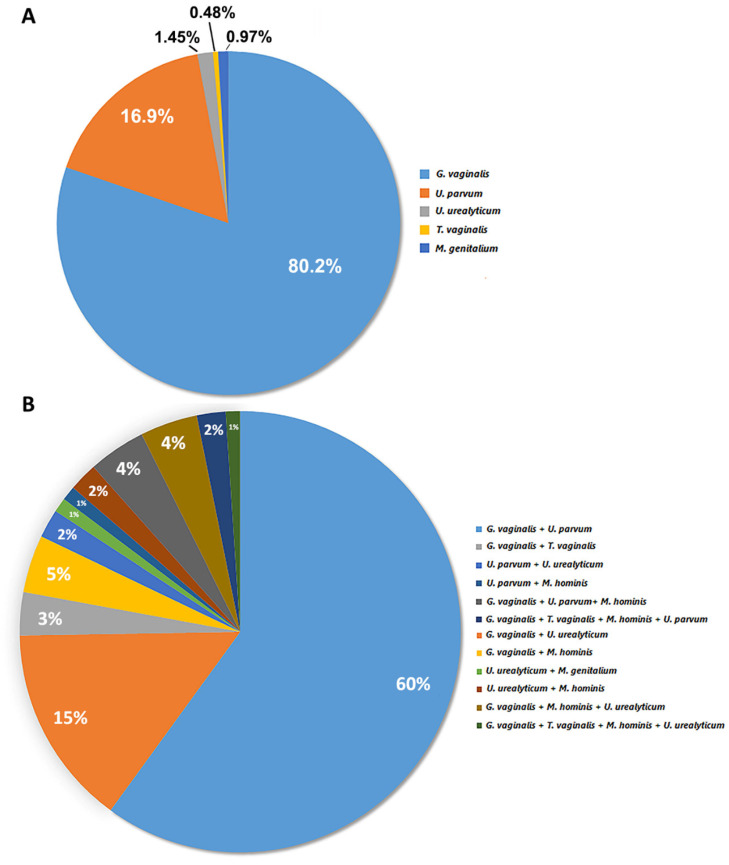
Distribution of single infection (**A**) and multiple infection (**B**) rates of genital microorganism in whole-positive cohort. In our study, among the subjects found positive for single infections (207/717) (Supplementary Materials Table S1), *G. vaginalis* and *U. parvum* represented the most common findings with a prevalence of 80.2% (166/207) and 16.9% (35/207), respectively (Figure 2A). Other identified microbial species included *U. urealyticum* (3/207; 1.45%), *M. genitalium* (2/207; 0.97%), and *T. vaginalis* (1/207; 0.48%) (Figure 2A).

The distribution of single and multiple infections for one or more genital microorganisms tested by mRT-PCR analysis is shown in Figure 2.

In our study, among the subjects found positive for single infections (207/717) (Supplementary Material: Table S1), *G. vaginalis* and *U. parvum* represented the most common findings with a prevalence of 80.2% (166/207) and 16.9% (35/207) respectively (Figure 2A). Other identified microbial species included *U. urealyticum* (3/207; 1.45%), *M. genitalium* (2/207; 0.97%) and *T. vaginalis* (1/207; 0.48%) (Figure 2A).

About the distribution of multiple infections, the double infection caused by *G. vaginalis* and *U. parvum* (57/95; 60%) resulted the most prevalent multiple infection, followed by *G. vaginalis* and *U. urealyticum* (14/95, 15%) (Table 2; Figure 2B); the remaining double infections represented together the 14% (13/95) of analyzed samples (Table 2; Figure 2B). Triple and quadruple infections showed a prevalence of 8% (8/95) and 3% (3/95), respectively (Table 2; Figure 2B). Among multiple infections, *G. vaginalis* was present in all the reported combinations (Figure 2B).

**Figure 3:**
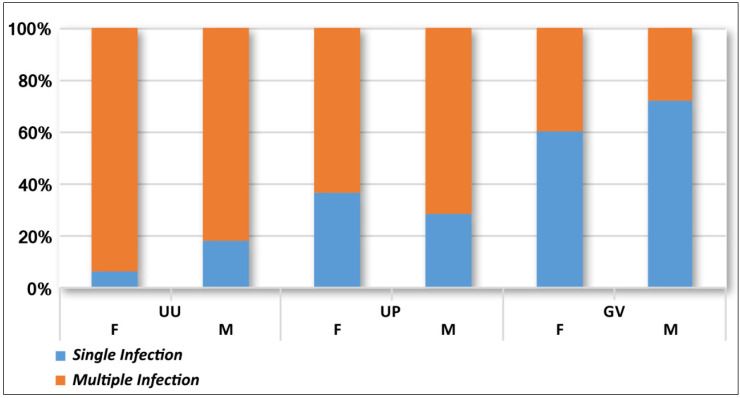
Prevalence of single and multiple infections due to *U. urealyticum* (UU), *U. parvum* (UP), and *G. vaginalis* (GV) in samples from female (F) and male (M) patients.

With regards to the pathogen distribution according to gender, Table 3 and Figure 3 highlight the patients’ prevalence of single and multiple infections in female and male genital tract specimens.

Our results showed that 52.34% (190/363) of women and 31.63% (112/354) of men were positive for at least one of the six genital microorganisms detected by mRT-PCR (Table 3).

Overall, among vaginal/endocervical swabs collected from women, 96/363 (26.44%) were positive for *G. vaginalis* DNA, 27/363 (7.43%) for *U. parvum* DNA, and 1/363 (0.27%) for *U. urealyticum* DNA, respectively (Table 3). With regards to the urine samples collected from men, *G. vaginalis* DNA was detected in 70/354 (19.77%) and *U. parvum* DNA in 8/354 (2.25%) (Table 3). For *U. parvum* infection, female subjects were 3-fold more exposed than male subjects (O.R. 3.47; CI: 1.55–7.76).

In the case of multiple infections, the prevalence was 18.18% (66/363) and 8.19% (29/354) in samples collected from women and men, respectively (Table 3), indicating that women were 2-fold more exposed than men (O.R. 2.48 CI: 1.56–3.95). Based on collected data, women presented a double risk to result positive to the analyzed genital microorganisms (O.R. 2.37; CI: 1.75–3.22) (Table 3).

Even though the present study evaluated the prevalence of a small number of genital microorganisms, considering the distribution of selected germs between single and multiple infections, *U. urealyticum* was found predominantly in multiple infections, regardless of gender, whereas, *G. vaginalis* was the prevailing agent of single infections (Figure 3).

## 4. Discussion

European epidemiological investigations revealed that the diagnosis and treatment of GIs still represent a major public health problem affecting both women and men worldwide. Globally, the increased frequency of GIs is thought to be due to changes in sexual behavior, such as contacts with a higher number of sexual partners, the lower average age of people engaging in sexual intercourse, and the inconsistency of condom use [29,30]. This is complicated by the fact that many GIs occur with no symptoms or with aspecific presentations, and that multiple infections are common. In this context, specific preventative and protective strategies associated with surveillance programs are urgently needed to face this problem. It is well known that a dysbiotic genital microenvironment with impaired bacterial tolerance and anti-inflammatory mechanisms influences the fertility, course of conception, and outcome of pregnancy, promoting the colonization and invasion of some opportunistic microorganisms or uropathogens that occur more often in women than men due to their anatomical features [17,31,32].

In this regard, the role of asymptomatic infection, the imbalance between potentially pathogenic microorganisms and the genital microbiota, and the immunological response to pathogens should be still investigated.

The aim of the present epidemiological study was to estimate the prevalence of GIs in the metropolitan area around Naples through the simultaneous detection of *U. urealyticum*/*U. parvum*; *M. hominis*/*M. genitalium*; and *T. vaginalis*/*G. vaginalis* by mRT-PCR.

Although the prevalence of genital pathogens shown in this report was greater among women (62.91%) compared to men (37.08%), *G. vaginalis* and *U. parvum* represented the most common findings with a prevalence of 80.2% and 16.9 %, respectively. Other identified microbial species included *U. urealyticum* (1.45%), *T. vaginalis* (0.48%), and *M. genitalium* (0.97%).

The evaluation of microbial co-infection prevalence showed that the most frequent associations detected in women and men were due to *G. vaginalis* and *U. parvum* (60%). Globally, female patients were significantly more affected than male patients in both single and multiple infections.

Considering the age of the analyzed subjects, the risk of GIs in the age group 30–39 years was higher than in the other age groups examined in our study, with a higher risk for both single and double infections.

In accordance with other published studies [22,33,34], our results showed that *G. vaginalis* and *U. parvum* were the most detected microorganisms in both women and men.

*G. vaginalis* was found in 40% of healthy women. Even if its role is yet to be fully elucidated, virulent strains of *G. vaginalis* are able to create microbial alterations by inducing bacterial vaginosis through adhesion to vaginal epithelial cells and biofilm formation [35,36]. Moreover, bacterial vaginosis due to *G. vaginalis* could also be associated to cervicitis, endometritis, salpingitis, and other relevant clinical implications [36].

*Ureaplasma* spp. were predominantly identified in the case of multiple infections. Although *Ureaplasma* spp. are considered part of the normal genital flora with an average colonization rate of 40–80% [37,38] they have been associated in a causal relationship with important clinical conditions such as infertility, non-gonococcal urethritis, and prostatitis [39]. An in vitro study showed the ability of *U. parvum* to form biofilm favoring its persistence, which contributes to the pathogenesis of chronic infections and facilitates mixed infection with other bacteria [40]. The role of *Ureaplasma* spp. in bacterial vaginosis remains highly controversial, since their presence has been demonstrated in a large proportion of women both with (65%) and without (48%) bacterial vaginosis [41]. Numerous clinical manifestations have been associated with *Ureaplasma* spp. Among the most notable is the role of *Ureaplasma* spp. in adverse pregnancy outcomes, such as chorioamnionitis and premature rupture of membranes leading to preterm birth [42,43,44]. Furthermore, recent literature data showed that the alteration of the normal genital flora with consequent co-infection of *Ureaplasma* and *G. vaginalis* represents a potential impairing factor of fertility [31].

It is well documented that some of the discussed microorganisms may be present as commensal flora in most of the sexually active people; due to their colonizing role, it is difficult to elucidate the pathogenic involvement of these agents, suggesting the need for epidemiological studies.

Although many efforts have been made to better diagnose the infections of genital tract in patients with symptoms [45], multiple molecular panels for the diagnosis of GIs could also be useful to screen the asymptomatic fertile population. In addition, since the association of multiple genital pathogens is one of the most frequent causes of treatment failure [46,47], the implementation of multiplex real-time PCR, as described here, provides the advantage of a simultaneous detection of several infectious agents in a single reaction, using the same specimen, reducing the sampling variability, ensuring the recognition of difficult cultivable germs, and offering to the clinicians the possibility to quickly prescribe appropriate antibiotics according to the results obtained. We are fully aware that there are some potential limitations to this study due to the limits of the molecular method compared to the reference culture method (inability to perform viable counts and antimicrobial susceptibility testing, requirement of expensive instruments), to the nature of retrospective studies, and to the possible presence of other microorganisms.

For these reasons, further studies are needed in order to develop new GI control strategies. The subjects enrolled in the present study were predominantly asymptomatic and had no personal STI history. If sexually active women and men with or without symptoms could be tested for the screening and diagnosis of GIs by multiplex molecular assays, it might be helpful to simultaneously detect several microorganisms.

In the present study, the significant presence of *G. vaginalis* and the observed association between *G. vaginalis* and *U. parvum* highlighted the importance of molecular screening as a valid tool for identifying infections in sexually active people which can be treated with the aim of reducing the risk of clinical complications.

## 5. Conclusions

In conclusion, our study provides an estimation of the prevalence of single and multiple infections caused by six common genital pathogens (*U. urealyticum*/*U. parvum*; *M. hominis*/*M. genitalium*, and *T. vaginalis*/*G. vaginalis*) among a large asymptomatic female and male population in a selected area of the Campania region in Southern Italy.

Overall, our epidemiological analysis reveals different patterns of infection between the genders and could help to identify groups at higher risk for clinical implications of GIs, underlining the need to implement a preventative screening strategy of genital pathogens.

## Figures and Tables

**Table 1:** Demographic characteristics of the subjects enrolled during the study period.

Demographic Characteristics of the Whole Population (717)	Patients Positive for Genital Pathogens Detection (302)	Patients Negative for Genital Pathogens Detection (415)	Chi Square Fisher’s Exact Test *p*-Value
*Women:* 363 (50.63%)	190/302 (62.91%)	173/415 (41.68%)	**<0.001**
*Men:* 354 (49.37%)	112/302 (37.08%)	242/415 (58.31%)	**<0.001**
*Women* (*years* ± SD)	36.91 ± 5.23	37.67 ± 10.41	0.375
*Men* (*years* ± SD)	38.69 ± 8.26	39.85 ± 8.98	0.243
*Mean age* (*years* ± SD)	37.59 ± 6.60	38.92 ± 9.66	0.145

Statistically significant *p*-values are indicated in bold.

**Table 2:** Analysis of multiple GIs detected in female and male specimens.

	Multiple GIs	SF + SM N (%)
Double infections	*G. vaginalis* + *U. parvum*	57 (60%)
	*G. vaginalis* + *U. urealyticum*	14 (15%)
	Other	13 (14%)
Triple infections	*G. vaginalis* + *U. parvum* + *M. hominis*	4 (4%)
	*G. vaginalis* + *U. urealyticum* + *M. hominis*	4 (4%)
Quadruple infections	*G. vaginalis* + *T. vaginalis*+ *M. hominis* + *U. parvum*	2 (2%)
	*G. vaginalis* + *T. vaginalis* + *M. hominis* + *U. urealyticum*	1 (1%)
Total samples		95 (100%)

SF: samples collected from female patients; SM: samples collected from male patients.

**Table 3:** Prevalence of GIs detected in female and male genital specimens.

Microorganisms	SF N (%)	SM N (%)	Chi-Square Fisher’s Exact Test
*U. urealyticum*	1 (0.27%)	2 (0.56%)	n.a.
*U. parvum*	27 (7.43%)	8 (2.25%)	**<0.001**
*G. vaginalis*	96 (26.44%)	70 (19.77%)	**<0.05**
Multiple Infection	66 (18.18%)	29 (8.19%)	**<0.001**
Positive	190 (52.34%)	112 (31.63%)	**<0.001**
Negative	173 (47.65%)	242 (68.36%)	
Total samples	363 (100%)	354 (100%)	

SF: samples collected from female patients; SM: samples collected from male patients. n.a.: not applicable. For *U. parvum* infection, women were 3-fold more exposed than men (O.R. 3.47; CI: 1.55–7.76). In the case of multiple infections, women were 2-fold more exposed than men (O.R. 2.48 CI: 1.56–3.95). Globally, women presented a double risk to be positive to the analyzed microorganisms (O.R. 2.37; CI: 1.75–3.22). Statistically significant *p*-values are indicated in bold.

## Data Availability

Not applicable.

## Supplementary Materials

The following supporting information can be downloaded at: https://www.mdpi.com/article/10.3390/diagnostics12081798/s1, Table S1: Distribution of genital infections between age study groups.
