# Supplementary material for: Molecular Epidemiology of Genital Infections in Campania Region: A Retrospective Study
Source: Diagnostics (Basel). 2022 Jul 25;12(8):1798. doi: 10.3390/diagnostics12081798 (PMC9394247; doi:10.3390/diagnostics12081798)
Supplement: Supplementary file 1 [file diagnostics-12-01798-s001.zip › diagnostics-1807317-supplementary.pdf]

Table S1. Distribution of genital infections between age study group.

**Age (years)**	**Number**	GIs **Positive (%)**	**Single Infection (%)**	**Double Infection (%)**	**Triple Infection (%)**	**Quadruple Infection (%)**
**<30**	68	19 (27.94%)	14 (20.59%)	4 (5.88%)	1 (1.47%)	0
**30-39**	362	174 (48.06%)	116 (32.04%)	52 (14.36%)	5 (1.38%)	1 (0.27%)
**40-49**	232	94 (40.52%)	65 (28.02%)	26 (11.20%)	2 (0.86%)	1 (0.43%)
**≥50**	55	15 (27.27%)	12 (21.81%)	2 (3.64%)	0	1 (1.82%)
Total	**717**	**302**	**207**	**84**	**8**	**3**
